# Low plasma progranulin levels in children with autism

**DOI:** 10.1186/1742-2094-8-111

**Published:** 2011-09-05

**Authors:** Laila Y AL-Ayadhi, Gehan A Mostafa

**Affiliations:** 1 Autism Research and Treatment Center, AL-Amodi Autism Research Chair, Department of Physiology, Faculty of Medicine, King Saud University, Riyadh, Saudi Arabia; 2 Department of Pediatrics, Faculty of Medicine, Ain Shams University, Cairo, Egypt

**Keywords:** Autism, autoimmunity, neutrophils, progranulin.

## Abstract

**Background:**

Autoimmunity to brain may play a pathogenic role in autism. In autoimmune disorders, the formation of antigen-antibody complexes triggers an inflammatory response by inducing the infiltration of neutrophils. Local administration of recombinant progranulin, which is an anti-inflammatory neurotrophic factor, potently inhibit neutrophilic inflammation in vivo, demonstrating that progranulin represents a crucial inflammation-suppressing mediator. We are the first to measure plasma progranulin levels in autism.

**Methods:**

Plasma levels of progranulin were measured, by ELISA, in 40 autistic patients, aged between 3 and 12 years, and 40 healthy-matched children.

**Results:**

Autistic children had significantly lower plasma progranulin levels, P = 0.001. Reduced plasma progranulin levels were found in 65% (26/40) of autistic children.

On the other hand, there was a non significant difference between plasma progranulin levels of children with mild to moderate autism and patients with severe autism, P = 0.11.

**Conclusions:**

Plasma progranulin levels were reduced in a subgroup of patients with autism. Progranulin insufficiency in some patients with autism may result in many years of reduced neutrotrophic support together with cumulative damage in association with dysregulated inflammation that may have a role in autism. However, these data should be treated with caution until further investigations are performed, with a larger subject population, to determine whether the decrease of plasma progranulin levels is a mere consequence of autism or has a pathogenic role in the disease. The role of progranulin therapy should also be studied in autism.

## Background

A possible role of abnormalities in the immune system in the pathogenesis of some neurologic disorders, including autism, was postulated [1,2]. Autoimmunity to the central nervous system may have a pathogenic role in autism [1]. This may be indicated by the presence of brain-specific auto-antibodies in some autistic children [3-9]. There is also an increase in the frequency of autoimmune disorders among autistic families [7,10-17]. In addition, there is a strong association between autism and the major histocompatibility complex for the null allele of C4B in class III region [15,18,19].

Neutrophils belong to the body's first line of cellular defense and respond quickly to tissue injury and invading microorganisms [20]. In autoimmune disorders, the underlying pathogenic mechanism is the formation of antigen-antibody complexes, so-called immune complexes (ICs), which trigger an inflammatory response by inducing the infiltration of neutrophils [21]. The subsequent stimulation of neutrophils by C3b-opsonized ICs results in the generation of reactive oxygen species (ROS) and the release of intracellularly stored proteases leading to tissue damage and inflammation [22]. It is therefore important to identify the mechanisms that control the activation of infiltrating neutrophils [23].

Progranulin, also known as proepithelin, acrogranin, or prostate cancer cell-derived growth factor, is a secreted glycosylated protein that undergoes proteolysis to generate seven mutually homologous 6-kD peptides, called granulins or epithelins [24,25]. Progranulin is released by a varaiety of cells and it is expressed by epithelial cells, macrophages, and neurons [24,26]. Progranulin is a growth factor implicated in tissue regeneration, tumorigenesis, and inflammation [24,27,28]. An in vitro study suggested that progranulin may function as a neurotrophic factor [29].

Progranulin was previously shown to directly inhibit adhesion-dependent neutrophil activation by suppressing the production of ROS and the release of neutrophil proteases in vitro. Local administration of recombinant progranulin potently inhibited neutrophilic inflammation in vivo, demonstrating that progranulin represents a crucial inflammation-suppressing mediator [27]. This anti-inflammatory activity is degraded by neutrophil-mediated proteolysis of progranulin to granulin peptides. In contrast, granulin peptides are strongly pro-inflammatory that enhance inflammation. Recent studies proposed progranulin as a regulator of the innate immune response, but the factors that control progranulin function are still poorly defined [23]. Progranulin inactivation might be involved in some autoimmune diseases such as small vessel vasculitis and lupus nephritis [30].

Since autism may be one of the pediatric autoimmune neuropsychiatric disorders, this study was the first to investigate the plasma levels of progranulin, which is an anti-inflammatory neurotrophic factor, in a group of autistic children.

## Methods

### Study population

This case-control study was conduced on 40 children who had classic-onset autism. The patients were fulfilling the criteria for the diagnosis of autism according to the 4th edition of the Diagnostic and Statistical Manual of Mental Disorders [31].

The autistic group comprised 32 males and 8 females. They were recruited from the Autism Research and Treatment Center, Faculty of Medicine, King Saud University, Riyadh, Saudi Arabia. Their ages ranged between 3 and 12 years (mean ± SD = 7.98 ± 2.59 years).

### Inclusion criteria

1- Patients who had no associated neurological diseases (such as cerebral palsy, tuberous sclerosis).

2- Patients who had no associated metabolic disorders (eg. Phenylketonuria) because these associated comorbidities with autism may influence the results of plasma progranulin levels.

3- Patients who were not receiving any medications.

The control group comprised 40 age- and sex-matched apparently healthy children. They included 33 males and 7 females. They were the healthy older siblings of the healthy children who attend the Well Baby Clinic, King Khalid University Hospital, Faculty of Medicine, King Saud University, Riyadh, Saudi Arabia for routine follow up of their growth parameters. The control children were not related to the children with autism, and demonstrated no clinical findings suggestive of immunological or neuropsychiatric disorders. Their ages ranged between 3 and 12 years (mean ± SD = 7.83 ± 2.64 years).

The local Ethical Committee of the Faculty of Medicine, King Saud University, Riyadh, Saudi Arabia, approved this study. In addition, an informed written consent of participation in the study was signed by the parents or the legal guardians of the studied subjects.

### Study measurements

#### Clinical evaluation of autistic patients

This was based on clinical history taking from the caregivers, clinical examination and neuropsychiatric assessment. In addition, the degree of the severity of autism was assessed by using the Childhood Autism Rating Scale (CARS) [32] which rates the child on a scale from one to four in each of fifteen areas (relating to people; emotional response; imitation; body use; object use; listening response; fear or nervousness; verbal communication; non-verbal communication; activity level; level and consistency of intellectual response; adaptation to change; visual response; taste, smell and touch response and general impressions). According to the scale, children who have scored 30-36 have mild to moderate autism (n = 12), while those with scores ranging between 37 and 60 points have severe autism (n = 28).

#### Assessment of plasma progranulin levels

We used the human progranulin ELISA kit (R&D Systems, Europe, Ltd.). This assay recognizes recombinant and natural human progranulin. No significant cross-reactivity or interference was observed. To increase accuracy, all samples were analyzed twice in two independent experiments to assess inter-assay variations and to ensure reproducibility of the observed results (P > 0.05).

### Statistical analysis

The results were analyzed by the commercially available software package (Statview, Abacus concepts, inc., Berkley, CA, USA). The data were non-parametric, thus they were presented as median and interquartile range (IQR). Mann-Whitney test was used for comparison between these data. Spearman's rank correlation coefficient "r" was used to determine the relationship between different variables. For all tests, a probability (p) of less than 0.05 was considered significant. Receiver operating characteristic (ROC) curve is a plotting of sensitivity versus 1-specificity at different cut-off values of the studied variable. The uppermost left point represents the best cut-off value (based on the highest sensitivity with the lowest false positive results of the studied marker) to differentiate the two groups under study. If the area under the curve (AUC) is > 0.5, this means that the variable is able to differentiate the two groups and the closer this area to 1 the better its differentiating ability. The best cut-off value of plasma progranulin was 83.5 ng/ml. AUC = 0.72, meaning that plasma progranulin was a good differentiating marker between patients and controls at this cut-off value.

## Results

**Figure 1:**
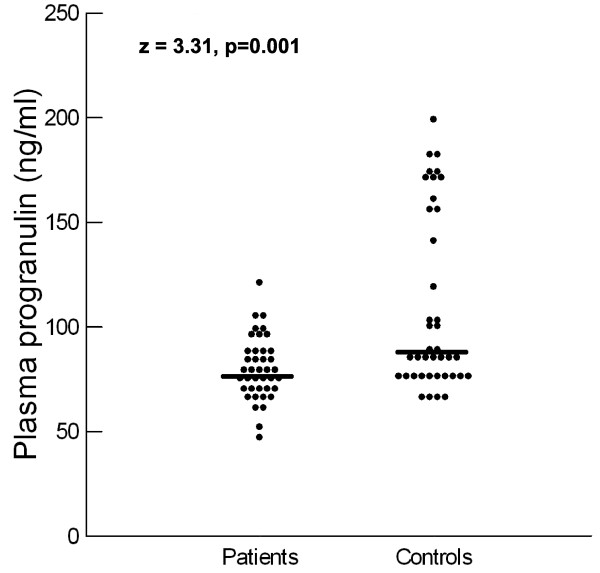
**Plasma progranulin levels in autistic patients and healthy children**. Median value for each group is shown by a horizontal bar.

Autistic children had significantly lower plasma progranulin levels [median (IQR) = 77.50 (19) ng/ml] than healthy controls [median (IQR) = 87.00 (81) ng/ml], P = 0.001 (Figure 1). Reduced plasma progranulin levels were found in 65% (26/40) of autistic children.

On the other hand, there was a non significant difference between plasma progranulin levels of children with mild to moderate autism and patients with severe autism, P = 0.11 (table 1). In addition, plasma progranulin levels of autistic patients had no significant correlations with CARS (P = 0.45).

**Table 1:** Plasma progranulin levels in relation to the degree of the severity of autism and the gender of the autistic patients

Patients with autism	Plasma progranulin (ng/ml) Median (IQR)	Z-value	P-value
Mild to moderate autism (n = 12)	74 (21)	0.52	0.61
Severe autism (n = 28)	79.5 (22)		
Male autistic patients (n = 32)	78 (19)	0.2	0.84
Female autistic patients (n = 8)	75 (34)		

There was a non significant difference between plasma progranulin levels of male and female autistic patients, P = 0.84, (table 1). Plasma progranulin levels of autistic patients had no significant correlations with the age of autistic children (P = 0.82).

## Discussion

Aetiology of autism presents many challenging issues and it has become an area of a significant controversy [33]. Autism may, in part, involve an autoimmune pathogenesis [1]. Neutrophils have a pathogenic role in autoimmunity [34]. Progranulin inhibits neutrophil activation and inflammatory activity, thus it represents a crucial inflammation-suppressing mediator [27].

In our series, autistic children had significantly lower plasma progranulin levels than healthy controls, P = 0.001. Reduced plasma progranulin levels were found in 65% (26/40) of autistic children. We could not trace data in the literature concerning progranulin levels in the blood of autistic children to compare our results. This study was the first to investigate plasma progranulin levels in autistic children.

Recently, much attention has been paid to the functional role of progranulin in the central nervous system because progranulin plays a key role in disease progression in neurodegenerative diseases [35-42]. Mutations in progranulin gene were identified as the cause of some forms of autosomal dominant tau-negative frontotemporal lobar degeneration (FTLD) [35,36], which is represented by severe atrophy in the frontal and temporal lobes of the brain. Therefore, haploinsufficiency with reduced progranulin-induced neuronal survival is thought to cause neurodegeneration [37]. Recent studies raise the possibility that FTLD may result in part from brain damage arising from the combination of dysregulated inflammation and heightened neuronal vulnerability as a result of reduced progranulin levels [43]. Progranulin level in the serum or the plasma is a reliable biomarker for diagnosis and early detection of FTLD caused by progranulin null mutations [44,45]. Progranulin protein is strongly reduced (up to 3.93-fold) both in plasma and CSF of affected and unaffected at risk subjects of families carrying the FTLD associated progranulin gene mutations. The dosage of plasma progranulin is a useful tool for a quick and inexpensive large-scale screening of carriers of progranulin mutations and for monitoring future treatments that might boost the level of this protein [46].

Progranulin has been implicated in inflammation, but its receptors remain unidentified. It was reported that progranulin bound directly to tumor necrosis factor receptors (TNFRs) and disturbed the TNFα-TNFR interaction. Progranulin-deficient mice were susceptible to collagen-induced arthritis, and administration of progranulin reversed inflammatory arthritis. Atsttrin, an engineered protein composed of three progranulin fragments, exhibited selective TNFR binding. Progranulin and Atsttrin prevented inflammation in multiple arthritis mouse models and inhibited TNFα-activated intracellular signaling. Collectively, these findings demonstrate that progranulin is a ligand of TNFR, an antagonist of TNFα signaling, and plays a critical role in the pathogenesis of inflammatory arthritis in mice [47,48]. In addition, macrophages from progranulin-deficient mice secrete less interleukin-10 and more inflammatory cytokines when exposed to bacterial lipopolysaccharide. Progranulin-deficient mice responded to infection with exaggerated inflammation. Ex vivo, progranulin-deficient hippocampal neurons were more vulnerable to metabolic stress. So, progranulin is a key regulator of inflammation and plays critical roles in both host defense and neuronal integrity [43].

Autism is recognized as having an inflammatory component. Post-mortem brain samples from patients with autism display neuroglial activation and an increase of the levels of inflammatory markers in cerebrospinal fluid, although little is known about the underlying molecular mechanisms [49]. Progranulin is a neuroprotective agent against neuroinflammation that often acts through the extracellular signal-regulated kinase and phopshatidylinositol-3-kinases pathways [50]. Thus, one should expect an increase in the production of the anti-inflammatory progranulin, which is a key regulator of inflammation, in autism. However, in the current study, the possible triggered inflammatory response in autism did not result in the increase of the production of the anti-inflammatory progranulin. This may be attributable to the presence of loss-of-function mutations in the progranulin gene in some patients with autism. However these data should be treated with a great caution till wide-scale studies investigating progranulin gene mutations in relation to plasma progranulin levels in autistic children are performed.

A recent study reported a significant elevation of CSF concentrations of progranulin in patients with relapsing-remitting multiple sclerosis (MS) during relapses compared with patients with relapsing-remitting MS during remissions and with non-inflammatory controls [51]. The elevation of CSF concentrations of progranulin in MS during relapse may be a local defense mechanism against inflammation by increasing the production of the anti-inflammatory progranulin to regulate the inflammatory process. Thus, the triggered inflammatory response in MS may result in increased progranulin levels in CSF of MS patients. This may indicate that progranulin gene mutations and progranulin deficiency may not have a role in MS.

Therefore in many inflammatory conditions such as MS, the increase of the production of the anti-inflammatory progranulin is expected to control the inflammatory process [51]. However, in some neuroinflammatory diseases, in which reduced progranulin levels may have a pathogenic role such as FTLD, mutations in progranulin gene with a subsequent progranulin deficiency may result in brain damage due to dysregulated inflammation secondary to reduced progranulin production [43]. This may be also the case in autism. A recent study reported the utility of prospective serum screening of progranulin as a surrogate diagnostic marker for progranulin gene mutations. It concluded that serum testing of progranulin is an accurate and cost effective means of predicting progranulin gene mutations in FTLD [52].

In the present work, there was a non significant difference between plasma progranulin levels of children with mild to moderate autism and patients with severe autism, P = 0.11. In addition, plasma progranulin levels of autistic patients had no significant correlations with CARS. These results may indicate that plasma progranulin levels were reduced in autistic patients regardless the degree of the disease severity.

A prominent role of progranulin in the regulation of inflammation was suggested by the discovery that neutrophil elastase and macrophage-derived secretory leukocyte protease inhibitor (SLPI) promote and prevent, respectively, the conversion of progranulin to granulin, and that recombinant progranulin inhibits neutrophil activation, whereas granulin promotes epithelial cell generation of neutrophil chemoattractants [27].

It is speculated that the autoimmune reaction to myelin in autism may result in the release of some neuronal antigens. These antigens, through the activation of inflammatory cells, could initiate autoimmune reactions with the production of brain-specific auto-antibodies in genetically susceptible individuals. These antibodies may cross the blood brain barrier and combine with brain tissue antigens forming immune complexes, thus further damaging the neurological tissue [53].

In autoimmune disorders, the formation of antigen-antibody immune complexes trigger an inflammatory response by inducing the infiltration of neutrophils [21] with subsequent ROS and the release of intracellularly stored proteases in neutrophils leading to tissue damage and inflammation [22]. In addition, neutrophil cells have been considered mainly as innate immune cells directed against microbial threats. Their serine proteases (neutrophil elastase and proteinase 3) are released at sites of inflammation and act as regulators of cell signaling and immune regulation. Neutrophil serine proteases act as alternative processing enzymes of pro-inflammatory cytokines IL-1β and IL-18 in vivo and modulate other inflammation-related control mechanisms such as progranulin inactivation in small vessel vasculitis and lupus nephritis [30]. We could not trace data in the literature regarding the neutrophil function in autistic patients, so we suggest that studies should be conducted to investigate neutrophil function in these patients. Since progranulin deficiency can promote the induction of autoimmunity through stimulation of neutrophil activation, the relationship between the low plasma progranulin levels and the induction of the production of brain-specific auto-antibodies in some autistic patients should also be studied.

In the central nervous system, progranulin is widely expressed during early neural development but later on, its expression becomes restricted to specific neuronal populations including cortical neurons in several layers, pyramidal cell layer and dentate gyrus of the hippocampus, ventromedial and arcuate nuclei of the hypothalamus, amygdale, and Purkinje cell layer in the cerebellum [26,37,54]. Recent studies suggest that progranulin is involved in neurotrophic activity and neuroinflammation [37,55]. An in vitro study reported that progranulin enhanced the neuronal survival and neurite length in cultured cortical and motor neurons. Interestingly, these effects were abolished by coadministration of SLPI, suggesiting that progranulin/granulin conversion plays a crucial role in their actions. In the central nervous system, SLPI has been known to be expressed in reactive astrocytes [56].

Therefore, progranulin insufficiency in some patients with autism may result in many years of reduced neutrotrophic support together with cumulative damage in association with dysregulated inflammation that may have a role in autism. However, we suggest that further studies, with a larger subject population, should be conducted to determine whether the decrease of plasma progranulin levels is a mere consequence of autism or has a pathogenic role in the disease.

## Conclusions

Plasma progranulin levels were reduced in a subgroup of patients with autism. Progranulin insufficiency in some patients with autism may result in many years of reduced neutrotrophic support together with cumulative damage in association with dysregulated inflammation that may have a role in autism. However, these data should be treated with caution until further investigations are performed, with a larger subject population, to determine whether the decrease of plasma progranulin levels is a mere consequence of autism or has a pathogenic role in the disease. In addition, wide-scale **s**tudies investigating progranulin gene mutations in relation to plasma progranulin levels and autoimmunity in autistic children are warranted. The role of progranulin therapy should also be studied in autism.

## Abbreviations

(AUC): area under the curve; (CARS): Childhood Autism Rating Scale; (CNS): central nervous system; (FTLD): frontotemporal lobar degeneration; (ICs): immune complexes; (IQR): interquartile range; (MS): multiple sclerosis; (ROC): Receiver operating characteristic curve; (ROS): reactive oxygen species; (SLPI): secretory leukocyte protease inhibitor; (TNF): tumor necrosis factor.

## Competing interests

The authors declare that they have no competing interests.

## Authors' contributions

Both authors designed, performed and wrote the research. In addition, both authors have read and approved the final manuscript.
